# Clinical measurements of aortic stiffness and peripheral resistance using a routine echocardiogram

**DOI:** 10.14814/phy2.70908

**Published:** 2026-05-12

**Authors:** Maggie Gudenkauf, Senthil Kumar, Noah D. Manring

**Affiliations:** ^1^ Mechanical and Aerospace Engineering University of Missouri Columbia Missouri USA; ^2^ University of Missouri Hospital Columbia Missouri USA

**Keywords:** aortic stiffness, echocardiogram, obesity paradox

## Abstract

Retrospective data for 259 patients is used to calculate aortic stiffness based on a new method which has been previously verified with in‐vitro and in‐hospital carotid‐femoral Pulse Wave Velocity (cfPWV) studies. For this group of patients, it is shown that the greatest variability within the hemodynamic record is the aortic stiffness, followed by the peripheral resistance – both present risk factors for cardiovascular disease. Our research shows that elevated stiffness is observed in both hypotensive and hypertensive patients, while the peripheral resistance increases monotonically with hypertensive grade. Our research also shows that stiffness and resistance *decrease* with increasing BMI which may indicate secondary causes for the obesity paradox documented in previous studies.

## 1 INTRODUCTION

### 1.1 Background

Research studies for arterial stiffness are gaining more relevance due to their importance in determining cardiovascular risk (Benetos et al., 2002; Kass et al., 2001; Lee & Kim, 2022; Mynard & Clarke, 2019; Shirwany & Zou, 2010; Spronck et al., 2022). The current “gold standard” for assessing arterial stiffness is the carotid‐femoral Pulse Wave Velocity (cfPWV) method. Reference values for cfPWV show that measurements above 13 m/s are linked to substantial risk for cardiovascular disease (Blacher et al., 1999; Laurent et al., 2001; Lemogoum et al., 2004; Mattace‐Raso et al., 2006; McEniery et al., 2005; Mitchell et al., 2010; Nichols, 2005; Segers et al., 2007; Soukup et al., 2022; Vlachopoulos et al., 2010). However, this test is not used in clinical practice (Baulmann et al., 2022; Mikael et al., 2017; Segers et al., 2009) and is not paid for by health insurance companies in the United States. Therefore, the aortic stiffness of a patient is not generally known by physicians who treat cardiovascular disease. Furthermore, the peripheral resistance is related to the mean arterial pressure and cardiac output. While this is not as elusive as stiffness, peripheral resistance is also rarely measured in the clinic but presents a risk factor for cardiovascular disease (Kähönen et al., 2020). By having access to both stiffness and resistance, clinicians are better equipped to treat cardiovascular disease. In this paper, we demonstrate a new method for assessing aortic stiffness and peripheral resistance by using five parameters that are routinely assessed in the clinic during an echocardiogram.

### 1.2 Literature review

#### 1.2.1 Stiffness surrogates

The literature presents various methods to assess cardiovascular stiffness. Some of these methods use the echocardiography‐Doppler method to calculate the PWV itself (Alhuzaimi et al., 2013; Escudero et al., 2018; Oh et al., 2017; Styczynski et al., 2016). Other studies use tonometer methods to determine the PWV, while adding echocardiographic imaging techniques for determining the waveforms (Ershova et al., 2016; Moco et al., 2017; Tavallali et al., 2018). Other measurements of arterial stiffness use an MRI for aortic stiffness; however, these MRI values are still used to calculate PWV as in previous studies (Hrabak‐Paar et al., 2020). Another study used a “dual impedance cardiography” method where electrodes were placed over a portion of the thorax and the calf, but the values measured were used, once again, for determining PWV (Scudder et al., 2021). Augmentation index is another research method for indirect measurement of arterial stiffness, but above the age of 60, the ability to accurately determine stiffness levels off, making it a poor predictor of stiffness for the elderly (Fok et al., 2014). The studies listed here are under research and signify the importance of having a more widely available method for measuring aortic stiffness in the clinic.

#### 1.2.2 Hypertension‐stiffness relationships

The interaction between hypertension and arterial stiffness has been referred to as the “stiffness‐hypertension dichotomy” (Gudenkauf, 2025; Safar et al., 2018; Zeng & Yang, 2024). The question is: What comes first, the hypertension or the stiffness? The answer seems to be that stiffening can precede high blood pressure, but high blood pressure can also result in arterial stiffening. For instance, a stiff aorta will produce a larger pulse pressure which results in high systolic pressures and lower diastolic pressures. In this case, elevated stiffness produces high systolic blood pressure. On the other hand, elevated mean blood pressure will stretch the aorta with a high volume of blood causing an increase in stiffness due to the nonlinear stress–strain relationship that exists for biological materials, like the aorta. This nonlinear relationship has been observed in our own experiments that have stretched a cow aorta with pressured water (Al‐Toki, 2020; Manring & Al‐Toki, 2020). In this case, high mean blood pressure has caused aortic stiffening.

#### 1.2.3 Drug therapies

There are clinical trials that have tested the effect of drug therapies for treating aortic stiffness (Brguljan Hitij et al., 2025; Cavero‐Redondo et al., 2024; Hamur et al., 2023; Jatic et al., 2019; Mack, 2024; Mahmud & Feely, 2005; Naqvi, 2023; O'Rourke, 1990; Protogerou et al., 2009; Radić et al., 2024; Song et al., 2024; Varol & Ozaydin, 2015; Zhang et al., 2024). One study by Jatic and colleagues investigated the different combinations of antihypertensive drug therapies in reducing stiffness and improving blood pressure management (Jatic et al., 2019). Overall, their study found that the use of antihypertensive medication decreased both hypertension and stiffening, with a greater reduction in the diastolic pressure rather than the systolic pressure. The use of antihypertensive drugs, such as angiotensin converting enzyme (ACE) inhibitors and beta blockers, has also been shown to help manage high arterial stiffness (Mallareddy et al., 2006; Varol & Ozaydin, 2015). Age and sex have been shown to influence the effectiveness of treating arterial stiffness with antihypertensive drugs. The PRECIOUS trial data was used to determine the differences between sex and age response when antihypertensive drug therapy used either a single pill or a combination pill treatment (Brguljan Hitij et al., 2025). The outcomes were measured by monitoring ambulatory blood pressure and arterial stiffness. The arterial stiffness was monitored using the cfPWV. Drug therapy was shown to reduce both blood pressure and stiffness. Overall, this study found that women responded better to drug intervention while older age groups showed less responsiveness compared to younger age groups.

#### 1.2.4 Obesity paradox

The obesity paradox describes the counterintuitive finding that overweight individuals with a particular disease may have better outcomes than their normal weight or underweight counterparts. This phenomenon is being discussed here as our current study shows healthier values for arterial stiffness and peripheral resistance for patients with a higher BMI. The review done by Drame and Godaert (Dramé & Godaert, 2023) selected 58 studies out of 2226 studies that were identified to find evidence for the obesity paradox. In conclusion, their work supports the existence of an obesity paradox, especially when comorbidities or acute medical problems are present.

#### 1.2.5 Our contribution

The new method presented in this paper uses existing clinical practice to assess cardiovascular stiffness and peripheral resistance, thereby making these assessments more readily available to physicians. Our new method for assessing aortic stiffness has been developed using a mathematical model based upon the conservation of blood mass within the cardiovascular system. This analysis has been published and verified using a cow aorta and a silicone‐rubber tube to conduct experiments and to show an in‐vitro correlation with a PWV test (Al‐Toki, 2020; Manring & Al‐Toki, 2020). Although this present publication does not include cfPWV in the retrospective data that is used, in previous research, the stiffness results were compared with cfPWV tests that were done on eight patients in the University of Missouri Hospital (Gudenkauf, 2025; Oliver et al., 2023, 2024). The correlation between the stiffness results and the cfPWV tests was shown to be good and much better than that of other surrogates like mean pressure, age, and pulse pressure.

### 1.3 Objectives

This research uses retrospective hospital data for patients who have undergone a physician‐prescribed echocardiogram to calculate aortic stiffness and peripheral resistance using equations that have been derived, presented, and verified in previous research (Al‐Toki, 2020; Gudenkauf, 2025; Manring & Al‐Toki, 2020; Oliver et al., 2023, 2024). Exclusion criteria for the study resulted in a cohort of 259 patients with relatively good health conditions, aside from varied blood pressure and obesity status. The first objective was to identify the greatest variability for the hemodynamic parameters of these patients. Our results show that aortic stiffness presents the greatest variability across the entire cohort of patients, followed by peripheral resistance. The second objective was to create statistical plots of stiffness and resistance as a function of hypertensive grade. Our results show that stiffness generally increases with hypertension, but that stiffness is also elevated for hypotensive patients. Peripheral resistance increases monotonically with hypertensive grade. The third objective was to create statistical plots of stiffness and resistance as a function of BMI. Our results show that both stiffness and resistance *decrease* with BMI, which suggests a secondary cause for the obesity paradox that has been observed in previous research (Dramé & Godaert, 2023).

### 1.4 Aortic stiffness, peripheral resistance, and previous work

A new calculation for aortic stiffness, *K*, has been derived in previous work, based on the principle of blood‐mass conservation within the aorta (Manring & Al‐Toki, 2020; Oliver et al., 2023, 2024):

$$K=\frac{P_{m}}{∆V}\frac{T}{T-T_{e}}\ln\left(\frac{P_{s}}{P_{d}}\right),$$

where $P_{m}$ is the mean arterial pressure, ∆V is the stroke volume of the left ventricle, *T* is the heartbeat period, $T_{e}$ is the left ventricle ejection period, *P*_*s*_ is the systolic pressure, and *P*_d_ is the diastolic pressure. All five of these parameters are measured during a routine echocardiogram. Peripheral resistance is a standard concept equal to the mean arterial pressure divided by cardiac output (Hall, 2015). Data from the echocardiogram may be used to calculate peripheral resistance as

$$R=\frac{P_{m}T}{∆V}.$$

In (Manring & Al‐Toki, 2020), we used an in‐vitro blood flow simulator to compare the stiffness calculation with the known stiffness of a silicone‐rubber tube. The device was also used to calculate the stiffness of an actual cow aorta harvested from a 1‐year‐old Holstein heifer. PWV measurements were made during these experiments and showed strong correlation with the stiffness calculation. In (Oliver et al., 2024), the authors compared the stiffness calculation with the cfPWV values for eight patients undergoing an echocardiogram in the University of Missouri Hospitals. This produced a coefficient‐of‐determination of 0.8499 which was much higher than comparisons made with other stiffness surrogates such as mean arterial pressure, pulse pressure, and age. This work was presented as a poster at the 2023 AHA annual meeting in Philadelphia (Oliver et al., 2023).

## 2 METHODS

Retrospective echocardiographic data for 259 patients was used in this study. Each patient was examined at the University of Missouri Hospital echocardiogram lab during October of 2024 for a physician prescribed, nonstress echocardiogram. Patients with the following characteristics were excluded from the study:

- Age less than eighteen.
- Heartrate above 100 bpm
- Structural abnormalities, such as left ventricle hypertrophy more than mild.
- Systolic or diastolic dysfunction more than mild, such as left ventricle systolic function with ejection fraction less than 50%
- Reduced right ventricle systolic function.
- Valvular disease containing any abnormality more than mild regurgitation (even trivial regurgitation for the aortic valve was excluded)
- Any form of valve stenosis or left ventricle outflow obstruction
- Heart diseases such as any form of cardiomyopathy, including hypertrophic cardiomyopathy or infiltrative cardiomyopathy with amyloid of the heart.
- Electrical abnormalities with any form of conduction delay or any rhythm abnormalities.
- Other rhythm abnormalities including premature ventricular contractions.
- Other abnormalities such as pericardial or pleural effusion
- Critically ill patients such as those on mechanical ventilatory support, those with pulmonary embolism, those undergoing shock treatment, etc.

These exclusion criteria produced a cohort of relatively healthy patients except for varied weight and blood pressure. For each of these patients we collected their age, sex, height, weight, systolic pressure, diastolic pressure, heartrate, left ventricle outflow tract diameter, and their left ventricle outflow tract velocity‐time‐integral directly from their echocardiographic patient report. From this information, we calculated body mass index (BMI), heartbeat period, cardiac output, and other hemodynamic parameters using standard methods. The left ventricle outflow tract cross‐sectional area was calculated using the left ventricle outflow tract diameter. Following standard echocardiogram methodology (Armstrong & Ryan, 2018), the left ventricle stroke volume was accurately assessed using Doppler echocardiography by estimating the blood flow across the left ventricle outflow tract from the cross‐sectional area. The Doppler signal assessed the blood velocity. Using this velocity with the cross‐sectional area of the orifice through which the blood flows allowed us to quantify the volumetric rate of blood from the left ventricle. During a routine echocardiogram, a pulse wave Doppler signal is measured in the left ventricle outflow tract. The Doppler signal is in units of velocity, and the area under the velocity‐time curve is in units of length. The left ventricle outflow tract is circular in shape and its area is calculated mathematically using the measured left ventricle outflow tract diameter in the parasternal long axis view by 2D echocardiography as a clinical standard. The velocity time integral, or stroke distance, is then multiplied by the area of the left ventricle outflow tract to yield the left ventricle stroke volume. The left ventricle ejection period equals the time duration of the pulse wave Doppler signal from the left ventricle outflow tract and is measured by drawing a line from the beginning to the end of the time scale. This procedure yields the left ventricle ejection period needed for our stiffness calculation. We then calculated the mean arterial pressure using the systolic and diastolic pressure, and the hemodynamic aortic stiffness and peripheral resistance using the equations previously presented in this paper.

Single blood pressure measurements were taken for each patient at the time of echocardiography. As a result, hypertensive classifications derived from our data may be influenced by situational variability and measurement error.

Data S1 contains the data for our patients. The gathered patient demographics are as follows. Of our 259 patients, 120 were inpatient and 139 were outpatient. There were 148 female patients and 111 male patients. The ages ranged from 18 years old to 90 years old, with an average of 54 years old. The BMI ranged from 15 kg/m^2^ to 77 kg/m^2^, with an average of 32 kg/m^2^. The average systolic pressure was 131 mm‐Hg, and the average diastolic pressure was 77 mm‐Hg.

We then sorted the data to be discussed in three ways: (1) into a single aggregate of 259 patients, (2) into the standard AHA hypertension groupings referred to as hypertensive grade, and (3) into standard CDC obesity groupings referred to as BMI grade. The AHA groupings include low blood pressure, normal, elevated, stage‐1, stage‐2, and crisis‐level hypertension. The CDC obesity groupings include underweight, healthy, overweight, class‐1, class‐2, and class‐3 (severe).

Box plots are used to display the distribution of stiffness and peripheral resistance. The boxplot is a compact way of displaying multiple aspects of the distribution of a continuous variable. The bottom and top of each box is positioned at the 25th and 75th percentiles, also called the first and third quartiles. The X symbol in the box is positioned at the mean value and the bar across the box is at the median value. The vertical lines extend to +1.5 times the interquartile range, a nonparametric measure spread approximately equal to +2 SD often used to describe normally distributed variables. Markers beyond the vertical limits indicate potentially atypical or outlying values.

## 3 RESULTS

### 3.1 Aggregate results

The aggregate results of the study for all 259 patients are shown in Table 1. As shown in the table, the aortic stiffness has the largest coefficient of variation across the entire cohort of patients. The peripheral resistance is next, and the lowest variation is observed in the mean arterial pressure though it is comparable to the variation of other hemodynamic parameters. From this table, we see that variations in stiffness and resistance play an important role in determining the blood‐pressure state of the patient. For this reason, we believe that these parameters should be measured routinely in the clinic to assist in developing treatment plans for cardiovascular disease.

**TABLE 1:** Aggregate results from retrospective data taken from 259 patients.

Parameter	Measured mean	Measured standard deviation	Measured coefficient of variation
Stiffness [mm‐Hg/mL]	1.273	0.517	0.406
Resistance [mm‐Hg‐s/mL]	1.248	0.375	0.300
Stroke volume [mL]	73.87	19.02	0.257
Heartbeat [bpm]	73.12	12.37	0.169
Ejection period [s]	0.315	0.047	0.148
Systolic pressure [mm‐Hg]	131.5	21.08	0.160
Mean arterial pressure [mm‐Hg]	103.7	14.79	0.143
Diastolic pressure [mm‐Hg]	77.71	11.51	0.148

### 3.2 Hypertensive grade results

**FIGURE 1:**
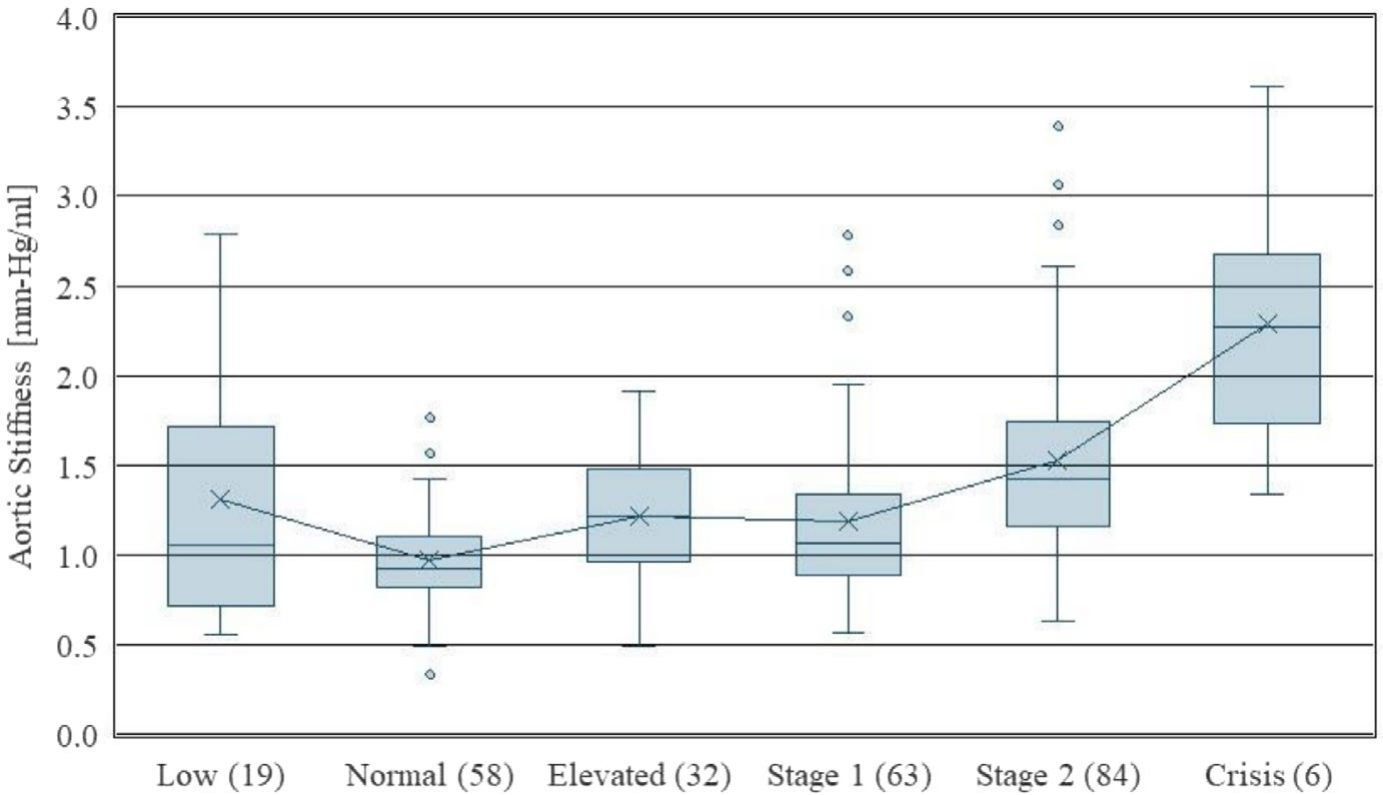
Aortic stiffness versus hypertensive grade. Numbers in parentheses indicate the number of patients in each classification.

Figure 1 displays the distribution of aortic stiffness by hypertensive grade, based upon a single blood pressure measurement during an echocardiogram. Definitions for each grade are given in Table 2. The boxplots in Figure 1 suggest that stiffness increases with the extremes of hypertension, is flat for mildly elevated cases (Elevated and Stage 1), and appears to be slightly higher for the low‐pressure subjects. Variability in stiffness is greater at both extremes of the hypertension spectrum, although this may be an artifact of small sample size for those categories. As a result, these findings should be interpreted with caution.

**TABLE 2:** AHA hypertensive grades used in this study (American Heart Association, 2025).

Grade	Criteria	No. of patients
Hypotensive, Low	SBP < 90 or DBP < 60	19
Normal	90 < SBP < 120 and 60 < DBP < 80	58
Elevated	120 < SBP < 129 and DBP < 80	32
Hypertensive, Stage‐1	130 < SBP < 139 or 80 < DBP < 89	63
Hypertensive, Stage‐2	140 < SBP < 179 or 90 < DBP < 119	84
Hypertensive, Crisis	180 < SBP or 120 < DBP	6

*Note*: According to these standards, three patients that are presented in the data of this research (Data S1) may be classified as both Hypotensive, Low and Hypertensive, Stage 1. This is because each of these patients present DBP <60 and 130 < SBP <139. These patient numbers are #65, #136, and #154 and in the subsequent analysis their data will be reported under both classifications, resulting in 262 data points for 259 patients.

Abbreviations: DBP, diastolic blood pressure; SBP, systolic blood pressure.

**FIGURE 2:**
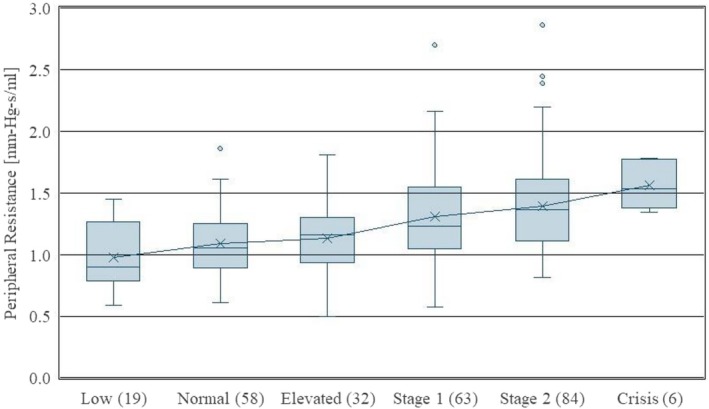
Peripheral resistance versus hypertensive grade. Numbers in parentheses indicate the number of patients in each classification.

Figure 2 displays a well‐defined trend of increasing mean and median peripheral resistance with hypertension grade. This figure shows that the peripheral resistance increases monotonically with the hypertensive classification, with very little spread in the data for hypertensive crisis patients. Again, the hypertensive grade was based upon a single blood pressure measurement taken during an echocardiogram.

### 3.3 BMI grade results

**FIGURE 3:**
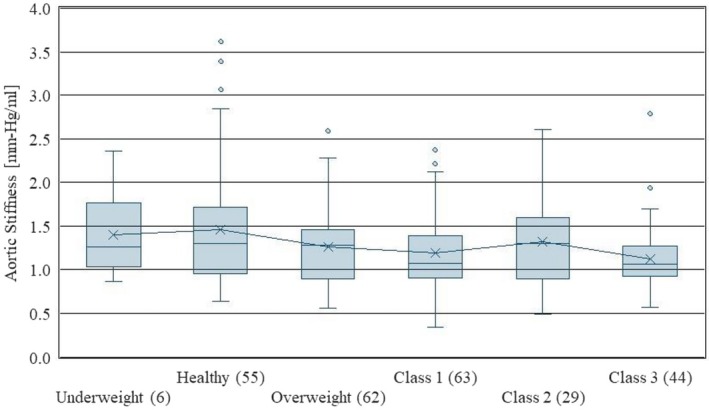
Aortic stiffness versus BMI grade. Numbers in parentheses indicate the number of patients in each classification.

**FIGURE 4:**
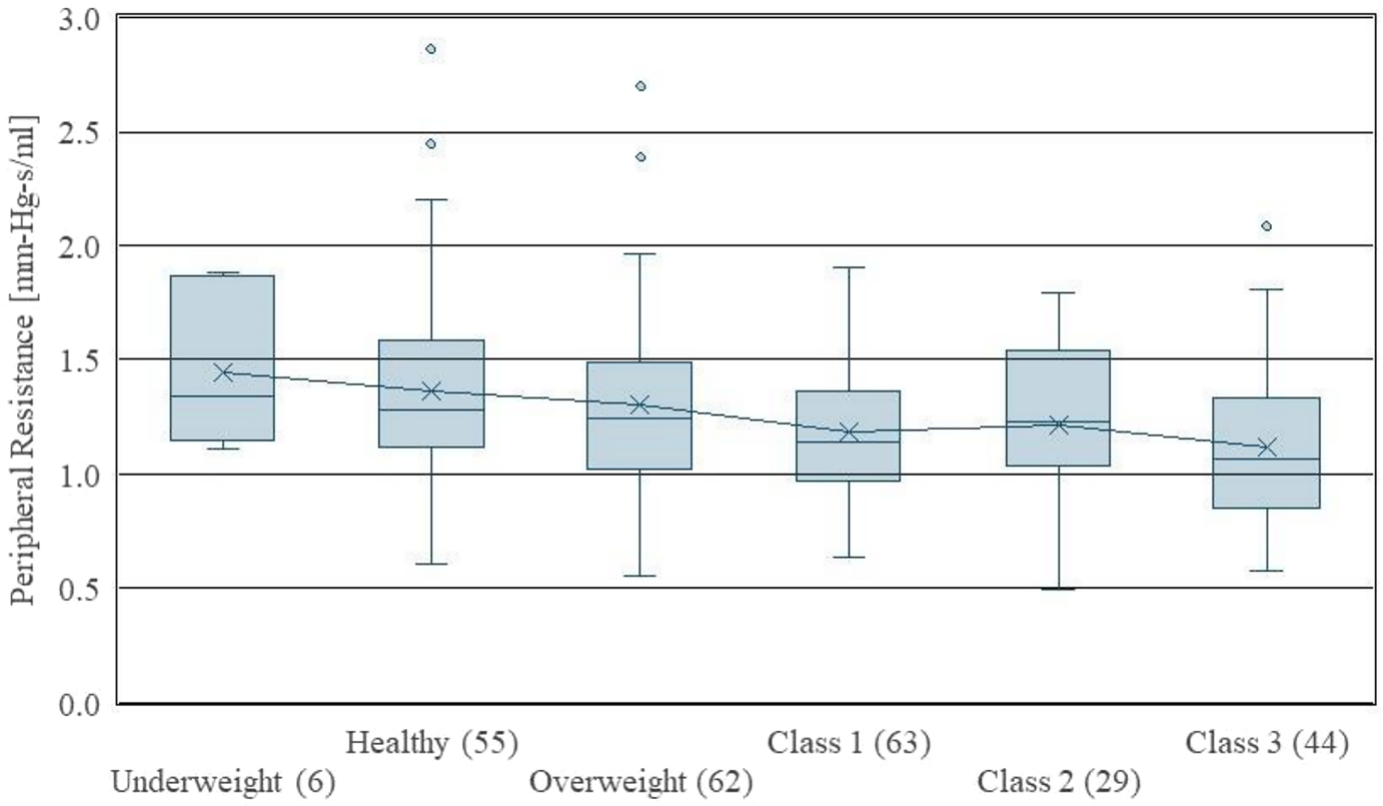
Peripheral resistance versus BMI grade. Numbers in parentheses indicate the number of patients in each classification.

Figure 3 shows a boxplot display of stiffness by BMI, for CDC BMI categories. Definitions for each grade are given in Table 3. Figure 4 shows a statistical plot of the cohort's peripheral resistance. As shown in Figure 3, the average aortic stiffness *decreases* with BMI grade. Figure 4 shows that average peripheral resistance decreases similarly. These are unexpected results as cardiovascular health is often connected to overweightness, and these results suggest that vascular health tends to improve with weight gain. The results suggest that the improved mechanical properties of stiffness and resistance may explain secondary causes for the obesity paradox (Dramé & Godaert, 2023).

**TABLE 3:** CDC, BMI grades used in this study (CDC, 2025).

Grade	Criteria	No. of patients
Underweight	BMI < 18.5	6
Healthy	18.5 < BMI < 25	55
Overweight	25 < BMI < 30	62
Class 1	30 < BMI < 35	63
Class 2	35 < BMI < 40	29
Class 3 (Severe)	40 < BMI	44

## 4 DISCUSSION AND CONCLUSIONS

From the aggregate data of 259 patients undergoing a routine echocardiogram at the University of Missouri Hospitals, it has been shown that the two hemodynamic parameters that have the greatest variability are the aortic stiffness and the peripheral resistance. It is well known that both parameters present risk factors for cardiovascular disease (Benetos et al., 2002; Blacher et al., 1999; Kähönen et al., 2020; Kass et al., 2001; Laurent et al., 2001; Lee & Kim, 2022; Lemogoum et al., 2004; Mattace‐Raso et al., 2006; McEniery et al., 2005; Mitchell et al., 2010; Mynard & Clarke, 2019; Nichols, 2005; Segers et al., 2007; Shirwany & Zou, 2010; Soukup et al., 2022; Spronck et al., 2022; Vlachopoulos et al., 2010). Due to the high variability of these parameters, they should be measured if a complete understanding of cardiovascular health is to be known; however, these two parameters are not typically measured in the clinic. This study provides a method to determine these parameters on a more regular basis using existing measurements that are taken during a routine echocardiogram.

As shown in Figure 1, aortic stiffness generally increases with hypertensive grade; however, this figure shows an elevated stiffness for patients that are hypotensive, which is an unexpected result. Both hypotensive and crisis hypertensive patients present a wide range of variability in their stiffness measurements. This figure suggests that treatment for elevated stiffness may need to be considered for both hypotensive and hypertensive patients. Treatments may include drug therapy such as ACE inhibitors, calcium channel blockers, or statins. For patients with overactive heart function, beta blockers may also help. Figure 2 shows that peripheral resistance increases monotonically with hypertensive grade. While stiffness and resistance are known risk factors for cardiovascular disease, our study does not show a direct correlation between these two risk factors. Patients with high stiffness may not present high resistance, and vice versa.

The results shown in Figure 3 and Figure 4 are highly unexpected and present an area needing future research to account for confounders such as age, sex, inpatient/outpatient status, blood pressure, heart rate, etc. Each figure shows a reduction in stiffness and resistance, respectively, as BMI increases. In other words, the results of this research indicated that greater obesity tends to increase the apparent health of the vasculature, which is counterintuitive. However, the obesity paradox may be at work here, which is known to show lower mortality rates for overweight people suffering from cardiovascular disease and cancer (Dramé & Godaert, 2023). Even so, alternative explanations for this result should also be more carefully considered. By showing lower stiffness and resistance numbers for obese patients, our research may indicate secondary causes for this paradoxical finding.

While various clinical implications have been suggested in the previous paragraphs, given the fact that we provide no outcome‐based validation of each treatment it is more appropriate to view the findings of this research as hypothesis‐generating rather than supportive of clinical implementation.

## AUTHOR CONTRIBUTIONS

**Noah D. Manring:** Conceptualization; formal analysis; supervision. **Senthil Kumar:** Conceptualization; data curation; investigation; methodology; supervision. **Maggie Gudenkauf:** Conceptualization; data curation; formal analysis; investigation; methodology; project administration; validation; visualization.

## FUNDING INFORMATION

The University of Missouri paid the regular salaries of the authors and made deidentified medical records available for retrospective analysis, as approved by the Institutional Review Board (Project Number 2019379).

## ETHICS STATEMENT

The manuscript is a retrospective case report that does not require ethics committee approval at the University of Missouri. Since the data have been deidentified, informed consent for anonymized publication was not required.

## Supporting information


**Data S1** Retrospective echocardiographic data for 259 patients.

## Data Availability

The deidentified data is presented in Data S1.
