# Supplementary material for: Clinical measurements of aortic stiffness and peripheral resistance using a routine echocardiogram
Source: Physiol Rep. 2026 May 12;14(9):e70908. doi: 10.14814/phy2.70908 (PMC13167701; doi:10.14814/phy2.70908)
Supplement: Supplementary file 1 — Data S1 Retrospective echocardiographic data for 259 patients. [file PHY2-14-e70908-s001.docx]

Supplementary File 1

Retrospective echocardiographic data for 259 patients.

| Patient # | Age [Years] | Sex  [M/F] | BMI  [kg/mm^2^] | Systolic Pressure  [mmHg] | Diastolic Pressure  [mmHg] | Heart Rate [bpm] | LV Ejection Period  [s] | LV Stroke Volume  [ml] |
| --- | --- | --- | --- | --- | --- | --- | --- | --- |
| 1 | 59 | F | 26.58 | 127 | 78 | 85 | 0.307 | 64.79 |
| 2 | 72 | M | 21.44 | 124 | 64 | 61 | 0.324 | 52.78 |
| 3 | 58 | F | 32.92 | 117 | 63 | 65 | 0.315 | 60.60 |
| 4 | 44 | F | 38.44 | 128 | 79 | 63 | 0.398 | 81.69 |
| 5 | 61 | F | 55.78 | 131 | 85 | 70 | 0.356 | 81.77 |
| 6 | 39 | F | 21.98 | 108 | 72 | 76 | 0.348 | 79.51 |
| 7 | 82 | M | 30.37 | 165 | 95 | 60 | 0.374 | 75.71 |
| 8 | 23 | M | 22.80 | 122 | 82 | 55 | 0.327 | 64.72 |
| 9 | 40 | F | 29.95 | 120 | 85 | 83 | 0.348 | 51.32 |
| 10 | 66 | F | 28.35 | 149 | 80 | 78 | 0.340 | 89.60 |
| 10 | 56 | F | 44.62 | 152 | 89 | 79 | 0.288 | 79.66 |
| 11 | 70 | M | 33.10 | 142 | 113 | 92 | 0.247 | 52.46 |
| 12 | 59 | M | 27.89 | 143 | 92 | 72 | 0.301 | 58.16 |
| 13 | 19 | F | 33.70 | 111 | 90 | 94 | 0.296 | 62.20 |
| 14 | 49 | F | 76.99 | 156 | 87 | 70 | 0.343 | 89.71 |
| 15 | 71 | F | 34.08 | 100 | 70 | 67 | 0.315 | 52.15 |
| 16 | 80 | F | 24.88 | 177 | 74 | 66 | 0.365 | 73.15 |
| 17 | 54 | F | 27.56 | 116 | 76 | 57 | 0.385 | 73.25 |
| 18 | 36 | F | 29.09 | 139 | 96 | 73 | 0.319 | 80.70 |
| 19 | 52 | M | 38.78 | 125 | 84 | 98 | 0.236 | 51.60 |
| 20 | 74 | F | 17.89 | 108 | 71 | 74 | 0.315 | 38.82 |
| 21 | 75 | F | 27.43 | 187 | 111 | 57 | 0.360 | 87.02 |
| 22 | 80 | F | 32.93 | 146 | 76 | 63 | 0.320 | 87.96 |
| 23 | 57 | F | 27.63 | 151 | 79 | 57 | 0.423 | 84.49 |
| 24 | 58 | F | 27.99 | 122 | 77 | 83 | 0.280 | 48.46 |
| 25 | 58 | M | 35.88 | 157 | 87 | 54 | 0.369 | 76.67 |
| 26 | 26 | F | 22.27 | 117 | 68 | 78 | 0.268 | 75.51 |
| 27 | 20 | F | 21.61 | 106 | 70 | 60 | 0.314 | 55.57 |
| 28 | 67 | F | 41.38 | 141 | 86 | 73 | 0.341 | 79.80 |
| 29 | 75 | M | 26.58 | 132 | 86 | 50 | 0.360 | 82.94 |
| 30 | 59 | F | 43.97 | 122 | 66 | 74 | 0.299 | 87.89 |
| 31 | 58 | F | 41.60 | 148 | 85 | 65 | 0.314 | 72.58 |
| 32 | 60 | M | 31.70 | 120 | 75 | 69 | 0.392 | 88.50 |
| 33 | 43 | F | 60.27 | 132 | 93 | 99 | 0.323 | 83.32 |
| 34 | 70 | M | 27.96 | 114 | 69 | 58 | 0.336 | 77.40 |
| 35 | 65 | F | 40.33 | 115 | 86 | 89 | 0.332 | 55.12 |
| 36 | 58 | M | 44.74 | 142 | 102 | 85 | 0.278 | 58.56 |
| 37 | 76 | M | 34.44 | 138 | 82 | 53 | 0.336 | 79.57 |
| 38 | 60 | F | 22.86 | 102 | 60 | 77 | 0.345 | 76.84 |
| 39 | 90 | F | 31.64 | 120 | 76 | 76 | 0.398 | 67.00 |
| 41 | 51 | M | 50.69 | 106 | 60 | 63 | 0.268 | 94.27 |
| 42 | 54 | M | 25.50 | 127 | 72 | 93 | 0.232 | 114.67 |
| 43 | 76 | M | 20.23 | 145 | 84 | 93 | 0.291 | 66.06 |
| 44 | 73 | M | 20.34 | 203 | 105 | 99 | 0.361 | 70.55 |
| 45 | 62 | F | 34.33 | 99 | 58 | 74 | 0.297 | 63.51 |
| 46 | 43 | M | 23.73 | 100 | 68 | 78 | 0.254 | 66.90 |
| 47 | 82 | F | 38.20 | 116 | 78 | 71 | 0.349 | 70.82 |
| 48 | 54 | F | 76.39 | 163 | 69 | 93 | 0.257 | 84.01 |
| 49 | 55 | M | 26.12 | 122 | 79 | 81 | 0.324 | 59.69 |
| 50 | 63 | M | 30.32 | 152 | 63 | 51 | 0.304 | 75.14 |
| 51 | 27 | F | 41.10 | 154 | 99 | 65 | 0.320 | 55.57 |
| 52 | 74 | F | 31.07 | 106 | 52 | 76 | 0.251 | 67.86 |
| 53 | 59 | M | 30.36 | 116 | 64 | 68 | 0.340 | 81.05 |
| 54 | 69 | M | 28.28 | 144 | 76 | 66 | 0.323 | 79.68 |
| 55 | 62 | F | 30.51 | 114 | 81 | 83 | 0.259 | 61.23 |
| 56 | 66 | M | 56.39 | 144 | 72 | 77 | 0.288 | 101.88 |
| 57 | 24 | M | 25.08 | 128 | 82 | 84 | 0.301 | 72.71 |
| 58 | 27 | M | 27.70 | 130 | 78 | 70 | 0.298 | 62.72 |
| 59 | 27 | F | 40.44 | 119 | 71 | 80 | 0.282 | 78.78 |
| 60 | 57 | M | 24.89 | 116 | 69 | 77 | 0.394 | 90.05 |
| 61 | 43 | F | 37.59 | 147 | 89 | 69 | 0.329 | 82.11 |
| 62 | 45 | M | 24.82 | 108 | 75 | 88 | 0.254 | 48.38 |
| 63 | 53 | F | 33.58 | 130 | 79 | 65 | 0.394 | 72.68 |
| 64 | 63 | F | 22.95 | 127 | 86 | 72 | 0.343 | 70.60 |
| 65 | 32 | F | 48.14 | 133 | 49 | 83 | 0.263 | 49.64 |
| 66 | 56 | F | 45.42 | 167 | 96 | 58 | 0.373 | 144.01 |
| 67 | 34 | F | 22.89 | 120 | 62 | 50 | 0.326 | 90.69 |
| 68 | 34 | F | 24.23 | 119 | 82 | 71 | 0.365 | 102.42 |
| 69 | 25 | F | 24.63 | 107 | 63 | 63 | 0.298 | 49.62 |
| 70 | 60 | F | 29.86 | 154 | 93 | 73 | 0.325 | 70.60 |
| 71 | 69 | M | 26.26 | 127 | 89 | 72 | 0.260 | 54.35 |
| 72 | 83 | F | 36.09 | 153 | 81 | 77 | 0.334 | 50.38 |
| 73 | 68 | M | 25.11 | 132 | 78 | 53 | 0.285 | 43.35 |
| 74 | 78 | F | 24.41 | 120 | 56 | 78 | 0.280 | 72.58 |
| 75 | 61 | M | 27.80 | 134 | 79 | 78 | 0.297 | 55.67 |
| 76 | 27 | F | 73.29 | 115 | 88 | 87 | 0.287 | 43.10 |
| 77 | 58 | F | 37.59 | 171 | 96 | 64 | 0.402 | 100.95 |
| 78 | 47 | F | 28.17 | 115 | 80 | 54 | 0.324 | 61.58 |
| 79 | 79 | F | 22.45 | 119 | 67 | 58 | 0.353 | 69.46 |
| 80 | 39 | M | 25.73 | 164 | 92 | 46 | 0.327 | 104.06 |
| 81 | 26 | F | 41.60 | 145 | 87 | 64 | 0.289 | 66.60 |
| 82 | 52 | M | 43.23 | 148 | 83 | 72 | 0.311 | 74.80 |
| 83 | 60 | F | 32.61 | 125 | 83 | 71 | 0.381 | 77.23 |
| 84 | 40 | M | 44.48 | 145 | 88 | 76 | 0.303 | 83.86 |
| 85 | 55 | F | 29.53 | 125 | 68 | 66 | 0.370 | 88.77 |
| 86 | 44 | F | 35.11 | 150 | 86 | 93 | 0.315 | 49.13 |
| 87 | 64 | M | 19.27 | 129 | 64 | 58 | 0.297 | 61.58 |
| 88 | 59 | M | 32.14 | 172 | 84 | 63 | 0.354 | 68.49 |
| 89 | 59 | F | 35.69 | 115 | 78 | 75 | 0.303 | 113.28 |
| 90 | 55 | M | 32.98 | 123 | 72 | 70 | 0.284 | 64.62 |
| 91 | 79 | M | 34.36 | 172 | 85 | 87 | 0.298 | 92.18 |
| 92 | 66 | F | 29.04 | 154 | 83 | 76 | 0.301 | 67.48 |
| 93 | 49 | F | 65.72 | 117 | 82 | 85 | 0.348 | 122.56 |
| 94 | 67 | M | 26.11 | 146 | 77 | 83 | 0.307 | 70.66 |
| 95 | 68 | F | 26.48 | 188 | 92 | 78 | 0.331 | 76.34 |
| 96 | 51 | M | 31.47 | 136 | 98 | 67 | 0.361 | 71.00 |
| 97 | 50 | M | 33.09 | 128 | 89 | 81 | 0.239 | 61.31 |
| 98 | 74 | F | 23.34 | 155 | 81 | 75 | 0.381 | 51.32 |
| 99 | 37 | M | 25.54 | 126 | 95 | 72 | 0.211 | 47.45 |
| 100 | 79 | F | 19.86 | 126 | 75 | 68 | 0.345 | 69.43 |
| 101 | 28 | M | 25.84 | 114 | 75 | 70 | 0.307 | 67.70 |
| 102 | 24 | F | 31.36 | 118 | 77 | 83 | 0.265 | 65.35 |
| 103 | 66 | M | 28.08 | 145 | 98 | 93 | 0.352 | 76.97 |
| 104 | 18 | M | 23.63 | 136 | 84 | 69 | 0.249 | 78.23 |
| 105 | 33 | F | 30.40 | 116 | 73 | 95 | 0.194 | 68.23 |
| 106 | 28 | F | 16.48 | 129 | 83 | 86 | 0.327 | 66.33 |
| 107 | 75 | M | 23.82 | 167 | 78 | 71 | 0.280 | 78.97 |
| 108 | 77 | M | 30.69 | 103 | 70 | 73 | 0.294 | 89.85 |
| 109 | 23 | F | 30.66 | 139 | 90 | 70 | 0.322 | 79.67 |
| 110 | 25 | F | 46.27 | 117 | 75 | 91 | 0.291 | 91.23 |
| 111 | 32 | M | 21.52 | 113 | 71 | 57 | 0.301 | 62.66 |
| 112 | 62 | F | 25.85 | 143 | 88 | 63 | 0.329 | 58.12 |
| 113 | 29 | F | 25.39 | 110 | 73 | 91 | 0.290 | 51.83 |
| 114 | 62 | M | 32.53 | 109 | 76 | 82 | 0.277 | 68.23 |
| 115 | 50 | F | 44.67 | 123 | 77 | 68 | 0.394 | 84.19 |
| 116 | 71 | F | 28.24 | 203 | 107 | 65 | 0.314 | 79.48 |
| 117 | 51 | M | 23.82 | 107 | 67 | 97 | 0.243 | 46.76 |
| 118 | 74 | M | 21.26 | 145 | 75 | 95 | 0.229 | 67.54 |
| 119 | 29 | F | 40.50 | 128 | 82 | 63 | 0.394 | 70.60 |
| 120 | 21 | F | 24.15 | 129 | 74 | 62 | 0.307 | 53.64 |
| 121 | 74 | M | 32.86 | 135 | 67 | 80 | 0.265 | 72.29 |
| 122 | 51 | M | 26.30 | 116 | 79 | 59 | 0.294 | 92.99 |
| 123 | 65 | M | 31.76 | 151 | 75 | 76 | 0.289 | 76.97 |
| 124 | 32 | F | 29.05 | 123 | 81 | 59 | 0.277 | 67.32 |
| 125 | 37 | F | 17.97 | 109 | 68 | 60 | 0.373 | 75.97 |
| 126 | 61 | F | 31.86 | 151 | 73 | 60 | 0.384 | 99.75 |
| 127 | 19 | F | 22.38 | 110 | 73 | 79 | 0.225 | 45.24 |
| 128 | 66 | M | 32.09 | 151 | 73 | 47 | 0.406 | 116.95 |
| 129 | 66 | M | 22.19 | 160 | 80 | 89 | 0.303 | 44.23 |
| 130 | 73 | M | 31.01 | 98 | 67 | 50 | 0.381 | 134.95 |
| 131 | 63 | M | 25.77 | 102 | 63 | 89 | 0.246 | 68.93 |
| 132 | 78 | F | 37.76 | 124 | 82 | 86 | 0.271 | 43.01 |
| 133 | 73 | M | 28.56 | 120 | 65 | 76 | 0.268 | 88.95 |
| 134 | 33 | F | 37.83 | 123 | 67 | 90 | 0.272 | 50.58 |
| 135 | 30 | M | 27.52 | 125 | 80 | 50 | 0.297 | 67.31 |
| 136 | 33 | M | 27.46 | 140 | 57 | 93 | 0.293 | 82.31 |
| 137 | 58 | M | 29.60 | 86 | 58 | 85 | 0.285 | 74.14 |
| 138 | 59 | F | 44.35 | 113 | 80 | 68 | 0.292 | 74.81 |
| 139 | 70 | M | 52.04 | 119 | 74 | 77 | 0.311 | 60.06 |
| 140 | 77 | F | 39.53 | 89 | 59 | 87 | 0.257 | 86.71 |
| 141 | 81 | M | 35.39 | 195 | 87 | 66 | 0.317 | 75.08 |
| 142 | 41 | F | 31.83 | 120 | 58 | 45 | 0.398 | 84.82 |
| 143 | 44 | F | 24.17 | 115 | 84 | 77 | 0.289 | 66.15 |
| 144 | 76 | M | 31.88 | 107 | 63 | 54 | 0.384 | 104.54 |
| 145 | 57 | F | 47.91 | 153 | 84 | 59 | 0.296 | 83.88 |
| 146 | 67 | M | 30.21 | 143 | 82 | 71 | 0.300 | 95.50 |
| 147 | 18 | F | 24.69 | 120 | 72 | 90 | 0.265 | 48.69 |
| 148 | 54 | F | 35.46 | 176 | 92 | 55 | 0.392 | 82.31 |
| 149 | 39 | M | 26.97 | 96 | 70 | 81 | 0.270 | 63.10 |
| 150 | 61 | F | 32.81 | 119 | 60 | 72 | 0.344 | 60.00 |
| 151 | 25 | F | 34.44 | 129 | 82 | 65 | 0.280 | 50.75 |
| 152 | 60 | M | 29.95 | 106 | 70 | 87 | 0.313 | 66.60 |
| 153 | 67 | F | 22.10 | 141 | 70 | 64 | 0.381 | 70.31 |
| 154 | 67 | M | 33.22 | 135 | 81 | 74 | 0.377 | 74.79 |
| 155 | 61 | M | 23.18 | 149 | 82 | 70 | 0.282 | 86.29 |
| 156 | 41 | F | 36.59 | 141 | 87 | 74 | 0.295 | 60.00 |
| 157 | 32 | F | 45.05 | 137 | 80 | 80 | 0.352 | 114.99 |
| 158 | 70 | F | 31.63 | 118 | 64 | 78 | 0.266 | 51.98 |
| 159 | 64 | F | 25.32 | 145 | 79 | 93 | 0.286 | 67.94 |
| 160 | 68 | F | 26.29 | 126 | 82 | 62 | 0.328 | 64.42 |
| 161 | 72 | M | 34.71 | 159 | 84 | 63 | 0.285 | 70.31 |
| 162 | 81 | M | 19.63 | 159 | 77 | 86 | 0.365 | 58.75 |
| 163 | 42 | F | 32.23 | 123 | 85 | 61 | 0.377 | 97.39 |
| 164 | 57 | F | 45.85 | 139 | 79 | 68 | 0.309 | 70.37 |
| 165 | 45 | F | 48.43 | 134 | 81 | 48 | 0.339 | 79.80 |
| 166 | 59 | M | 27.12 | 176 | 96 | 98 | 0.273 | 69.94 |
| 167 | 76 | M | 27.19 | 129 | 66 | 79 | 0.370 | 91.23 |
| 168 | 65 | F | 36.93 | 104 | 61 | 56 | 0.339 | 73.08 |
| 169 | 59 | F | 26.57 | 147 | 91 | 70 | 0.352 | 72.02 |
| 170 | 21 | F | 34.48 | 137 | 80 | 90 | 0.257 | 81.39 |
| 171 | 34 | F | 26.57 | 129 | 86 | 84 | 0.297 | 58.19 |
| 172 | 43 | M | 31.56 | 127 | 88 | 59 | 0.298 | 91.23 |
| 173 | 66 | M | 47.97 | 146 | 80 | 72 | 0.361 | 112.64 |
| 174 | 26 | F | 42.28 | 129 | 73 | 79 | 0.298 | 75.40 |
| 175 | 50 | M | 32.94 | 131 | 83 | 69 | 0.263 | 64.77 |
| 176 | 66 | F | 31.28 | 134 | 70 | 48 | 0.369 | 67.54 |
| 177 | 68 | M | 30.20 | 109 | 66 | 77 | 0.327 | 80.52 |
| 178 | 62 | F | 24.51 | 139 | 83 | 74 | 0.311 | 54.46 |
| 179 | 71 | M | 29.21 | 151 | 106 | 95 | 0.289 | 87.67 |
| 180 | 47 | F | 51.68 | 142 | 91 | 53 | 0.424 | 122.61 |
| 181 | 39 | F | 22.71 | 102 | 64 | 61 | 0.390 | 85.96 |
| 182 | 42 | M | 47.79 | 157 | 86 | 77 | 0.361 | 113.28 |
| 183 | 37 | M | 34.69 | 122 | 74 | 57 | 0.277 | 134.33 |
| 184 | 59 | M | 30.77 | 118 | 68 | 65 | 0.420 | 108.34 |
| 185 | 81 | M | 31.56 | 116 | 65 | 65 | 0.263 | 77.91 |
| 186 | 20 | M | 19.80 | 115 | 65 | 92 | 0.273 | 49.53 |
| 187 | 77 | M | 32.87 | 112 | 66 | 93 | 0.292 | 89.71 |
| 188 | 72 | F | 33.99 | 156 | 83 | 84 | 0.369 | 79.80 |
| 189 | 84 | F | 25.10 | 107 | 64 | 65 | 0.328 | 71.94 |
| 190 | 35 | F | 54.56 | 98 | 60 | 66 | 0.327 | 83.88 |
| 191 | 75 | F | 28.34 | 126 | 82 | 57 | 0.336 | 76.55 |
| 192 | 65 | F | 41.19 | 126 | 73 | 73 | 0.291 | 88.28 |
| 193 | 34 | M | 35.71 | 124 | 68 | 85 | 0.208 | 50.89 |
| 194 | 57 | F | 31.64 | 99 | 60 | 65 | 0.370 | 92.48 |
| 195 | 53 | F | 49.07 | 130 | 76 | 89 | 0.256 | 86.24 |
| 196 | 82 | M | 38.74 | 132 | 67 | 73 | 0.381 | 71.35 |
| 197 | 29 | M | 28.05 | 123 | 75 | 55 | 0.345 | 73.43 |
| 198 | 30 | F | 33.45 | 112 | 66 | 72 | 0.277 | 64.40 |
| 199 | 60 | F | 16.63 | 180 | 95 | 81 | 0.365 | 73.20 |
| 200 | 63 | M | 20.21 | 133 | 81 | 62 | 0.253 | 55.76 |
| 201 | 52 | M | 35.93 | 168 | 95 | 67 | 0.361 | 101.88 |
| 202 | 49 | M | 35.44 | 129 | 77 | 87 | 0.348 | 143.34 |
| 203 | 52 | M | 43.23 | 129 | 83 | 74 | 0.217 | 81.23 |
| 204 | 61 | F | 37.60 | 143 | 79 | 68 | 0.369 | 100.85 |
| 205 | 69 | M | 32.55 | 132 | 82 | 61 | 0.332 | 124.64 |
| 206 | 59 | M | 34.98 | 112 | 66 | 66 | 0.284 | 80.36 |
| 207 | 65 | F | 20.36 | 149 | 89 | 89 | 0.243 | 58.43 |
| 208 | 59 | M | 42.04 | 147 | 94 | 62 | 0.352 | 84.51 |
| 209 | 46 | M | 36.73 | 108 | 73 | 79 | 0.274 | 112.68 |
| 210 | 19 | M | 22.15 | 127 | 66 | 68 | 0.263 | 58.41 |
| 211 | 70 | F | 20.35 | 171 | 79 | 57 | 0.187 | 44.23 |
| 212 | 75 | M | 35.39 | 141 | 92 | 77 | 0.313 | 52.46 |
| 213 | 35 | M | 54.94 | 150 | 110 | 97 | 0.232 | 75.39 |
| 214 | 79 | M | 15.49 | 108 | 74 | 67 | 0.303 | 43.10 |
| 215 | 35 | F | 20.60 | 95 | 64 | 75 | 0.292 | 61.24 |
| 216 | 73 | M | 28.66 | 134 | 67 | 78 | 0.289 | 82.94 |
| 217 | 57 | F | 33.23 | 96 | 49 | 91 | 0.250 | 32.61 |
| 218 | 53 | M | 18.82 | 91 | 55 | 95 | 0.308 | 76.03 |
| 219 | 32 | F | 25.06 | 96 | 60 | 72 | 0.301 | 63.38 |
| 220 | 77 | F | 27.39 | 121 | 64 | 76 | 0.263 | 61.65 |
| 221 | 18 | M | 25.52 | 114 | 69 | 49 | 0.365 | 115.18 |
| 222 | 32 | F | 33.47 | 116 | 77 | 94 | 0.340 | 61.89 |
| 223 | 31 | F | 21.63 | 113 | 82 | 76 | 0.29 | 65.35 |
| 224 | 68 | F | 29.72 | 126 | 74 | 82 | 0.264 | 68.49 |
| 225 | 68 | M | 31.31 | 124 | 51 | 49 | 0.364 | 76.65 |
| 226 | 26 | F | 24.10 | 120 | 77 | 82 | 0.304 | 77.60 |
| 227 | 53 | F | 37.74 | 145 | 84 | 67 | 0.390 | 81.37 |
| 228 | 54 | M | 20.38 | 164 | 87 | 72 | 0.277 | 46.81 |
| 229 | 57 | F | 39.06 | 129 | 84 | 69 | 0.363 | 94.88 |
| 230 | 53 | M | 40.72 | 147 | 88 | 70 | 0.311 | 55.29 |
| 231 | 54 | F | 36.57 | 115 | 74 | 96 | 0.278 | 90.16 |
| 232 | 31 | F | 29.05 | 115 | 75 | 87 | 0.293 | 62.00 |
| 233 | 76 | F | 22.24 | 138 | 78 | 70 | 0.319 | 54.72 |
| 234 | 79 | F | 30.45 | 151 | 83 | 66 | 0.381 | 56.24 |
| 235 | 45 | F | 21.03 | 128 | 70 | 78 | 0.298 | 71.63 |
| 236 | 21 | F | 18.95 | 121 | 75 | 71 | 0.323 | 70.03 |
| 237 | 73 | F | 23.84 | 133 | 73 | 71 | 0.289 | 39.70 |
| 238 | 63 | M | 30.54 | 146 | 75 | 71 | 0.320 | 59.00 |
| 239 | 61 | F | 34.75 | 144 | 91 | 81 | 0.266 | 67.48 |
| 240 | 70 | M | 29.41 | 162 | 85 | 70 | 0.443 | 76.97 |
| 241 | 77 | M | 26.00 | 114 | 55 | 80 | 0.270 | 75.16 |
| 242 | 71 | F | 31.47 | 169 | 73 | 83 | 0.336 | 85.14 |
| 243 | 49 | M | 28.32 | 159 | 89 | 55 | 0.370 | 55.86 |
| 244 | 23 | F | 24.98 | 112 | 74 | 71 | 0.365 | 69.74 |
| 245 | 34 | F | 47.71 | 138 | 75 | 78 | 0.332 | 78.54 |
| 246 | 67 | F | 34.53 | 158 | 76 | 73 | 0.293 | 65.46 |
| 247 | 50 | F | 23.08 | 143 | 99 | 77 | 0.240 | 38.28 |
| 248 | 54 | M | 22.67 | 147 | 101 | 84 | 0.356 | 64.09 |
| 249 | 40 | F | 42.51 | 139 | 87 | 81 | 0.328 | 89.03 |
| 250 | 18 | F | 20.08 | 112 | 64 | 70 | 0.276 | 53.72 |
| 251 | 72 | M | 36.26 | 132 | 74 | 64 | 0.301 | 81.68 |
| 252 | 47 | F | 18.01 | 113 | 79 | 82 | 0.311 | 54.66 |
| 253 | 70 | F | 37.20 | 132 | 82 | 78 | 0.336 | 66.60 |
| 254 | 58 | F | 27.99 | 133 | 58 | 85 | 0.301 | 52.68 |
| 255 | 64 | F | 38.27 | 126 | 80 | 78 | 0.348 | 71.63 |
| 256 | 33 | F | 21.79 | 110 | 72 | 56 | 0.398 | 81.68 |
| 257 | 40 | M | 25.32 | 175 | 93 | 64 | 0.254 | 62.72 |
| 258 | 62 | F | 42.84 | 117 | 71 | 81 | 0.322 | 97.39 |
| 259 | 37 | F | 30.62 | 134 | 78 | 92 | 0.294 | 78.28 |
